# Supplementary material for: Snail reprograms glucose metabolism by repressing phosphofructokinase PFKP allowing cancer cell survival under metabolic stress
Source: Nat Commun. 2017 Feb 8;8:14374. doi: 10.1038/ncomms14374 (PMC5309788; doi:10.1038/ncomms14374)
Supplement: Supplementary Information — Supplementary Figures. [file ncomms14374-s1.pdf]

Supplementary Figure 1

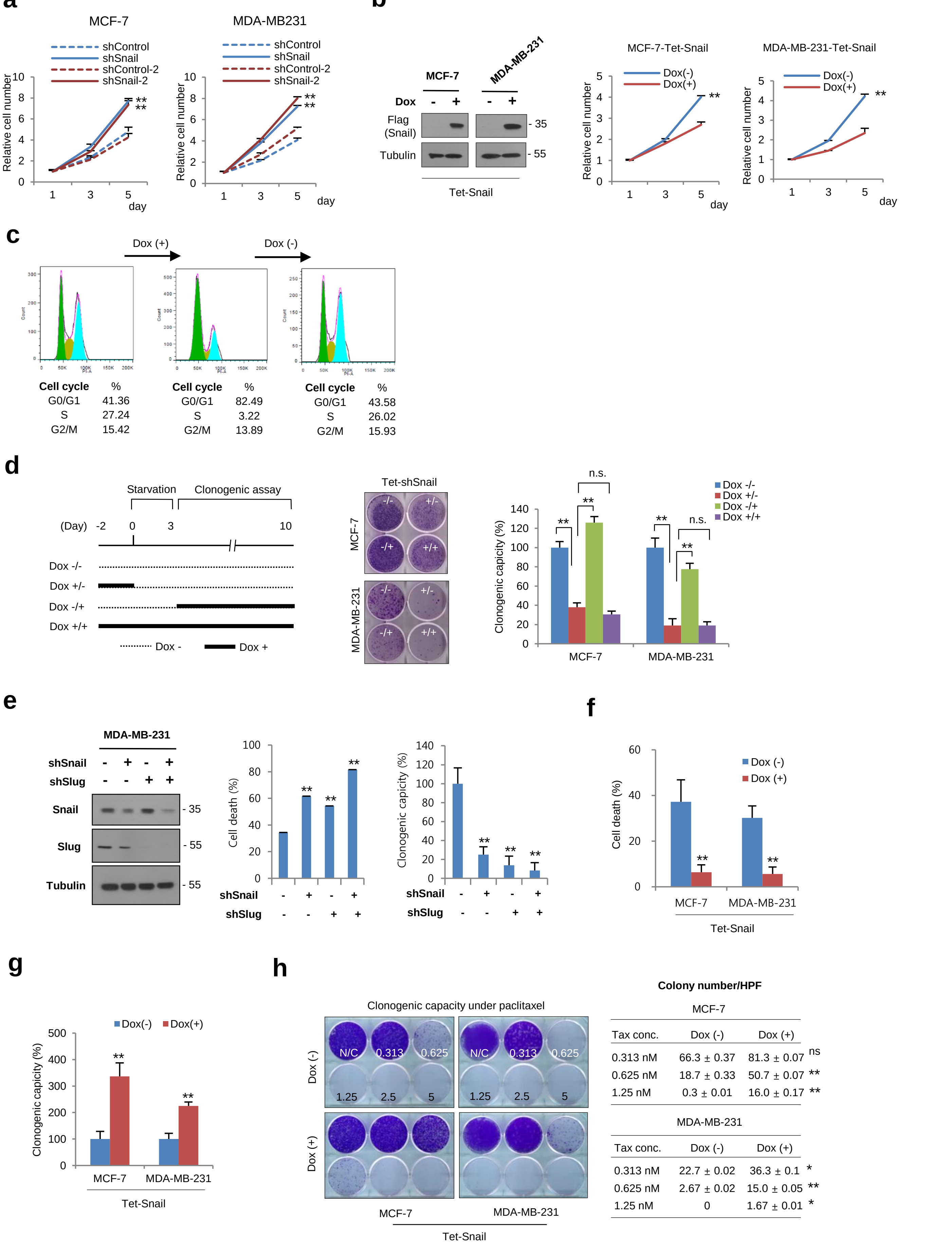

Supplementary Figure 1 EMT inducer potentiates cancer cell survival under metabolic stress. (**a**) Knock-down of Snail (shSnail) increased cell proliferation under normal culture condition in MCF-7 and MDA-MB-231 breast cancer cells. Cell proliferation was determined by MTT assay. (**b**) Immunoblot (left) and cell growth (right) expressing doxycyclin inducible Snail. Representative blots are shown from at least two independent experiments. Cell proliferation was determined by MTT assay. (**c**) The cellular DNA contents were analyzed with flow cytometer after propidium iodide staining (0.05 mg/ml in PBS). Doxycycline (3 μg/ml) was transiently treated for 48 h (Dox +) followed by refreshment of normal culture medium for 48 h (Dox-) in MDA-MB-231 cells. (**d**) Experimental design of clonogenic assay to validate the Snail requirement for immediate response to metabolic stress (left). The starved breast cancer cells were cultured with normal medium without (Dox-) or with (Dox +) doxycyline to induce Snail shRNA, and clonogenic capicity was visualized (middle and right). (**e**) Knock-down of Slug as well as Snail inhibits cell survival under metabolic stress. The MDA-MB-231 cells were transduced lentivirus expressing shRNA for Snail or Slug, or a combination of both shRNA. Immunoblot analysis (left), cell death (middle), and clonogenic capacity (right) were measured. (**f**) Cell death under glucose-deprived condition of cancer cells expressing inducible-Snail (Dox+). (**g**) Clonogenic capacity under glucose-deprived condition followed by refreshment of normal culture medium. (**h**) Clonogenic survival of breast cancer cells against paclitaxel treatment as indicated by concentration. The Snail was induced with doxycycline (Dox +) for 48 h prior to paclitaxel treatment (left panels). The colony number was determined by stereomicroscopic examination under high power field (HPF) as described in Methods (right panels). Data are means ± s.d. from n = 3 (**b**, **e**, **g**, **h**) or n = 5 (**a**, **d**, **f**) independent experiments. Statistical significances compared to control was denoted as *, $P < 0.05$; **, $P < 0.01$; n.s., not significant by a two-tailed Student's t-test. Experiments (**a**, **b**, **f**, **g**) are representative and were replicated from at least two independent experiments. Unprocessed original scans of blots are shown in Supplementary Fig. 11.

Supplementary Figure 2

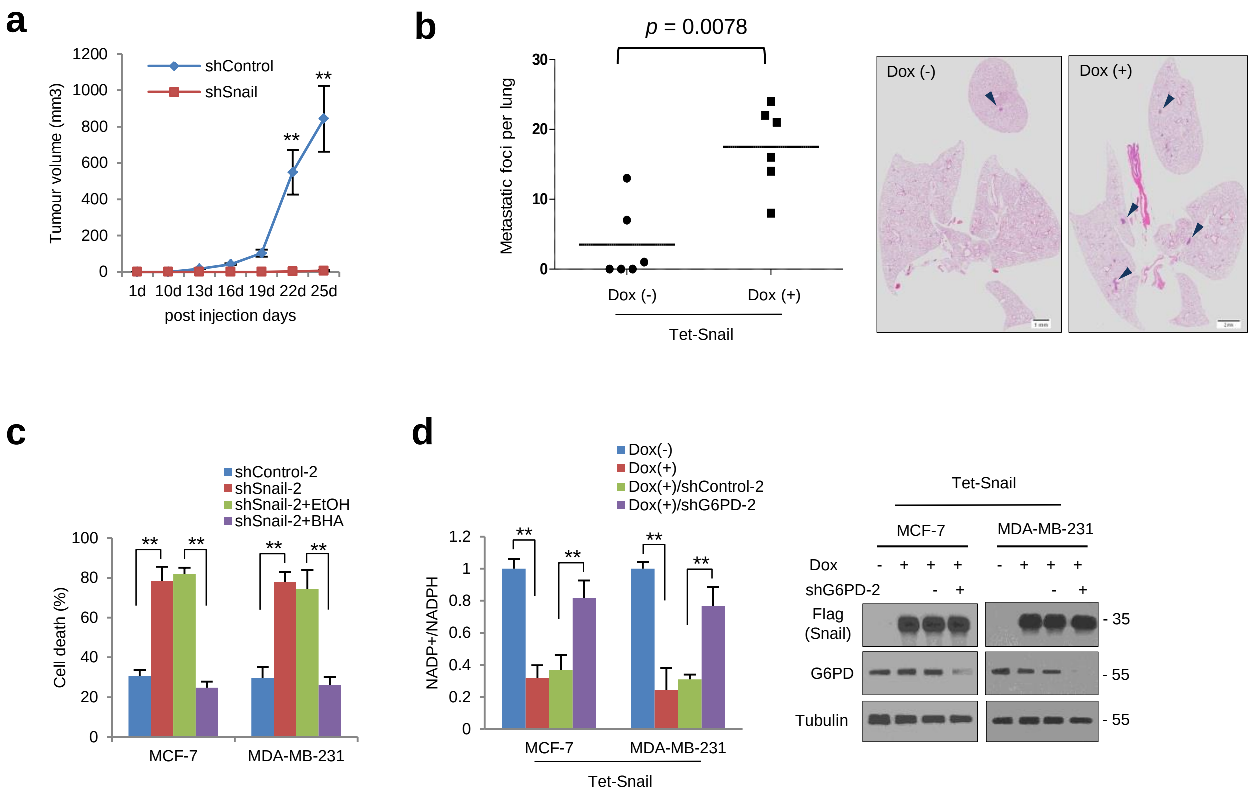

Supplementary Figure 2 Snail abundance regulates ROS level and is required for in vivo tumour initiation and metastasis. (**a**) MDA-MB-231 cells (1 X 10^6) expressing shControl (n = 6) or shSnail (n = 6) were injected orthotopically into the mammary fat pads of nude mice. Tumour initiation was monitored biweekly. Results are shown as means and s.e.m. Two asterisks, $P < 0.01$ compared to the control by Mann-Whitney test. (**b**) Lung metastasis by tail vein xenograft of MDA-MB-231-D3H2LN cells. 1 x 10^5 cells either of control (Dox-, n = 6) or overexpression of Snail (Dox+, n = 6) were inoculated intravenously into athymic nude mice. The number of lung metastatic nodules at day 28 was counted under microscopic examination (left). Statistical significance was determined by Mann-Whitney test. Whole-field images of representative lungs that showed median metastatic value for each group (right). Arrows indicate metastatic tumour foci in mouse lung; scale bars are 1 mm. (**c**) Antioxidant BHA (100 µM) treatment rescued cell death induced by glucose deprivation of independent set of Snail knock-downed cells (shSnail-2). (**d**) Snail was induced by treatment of doxycycline (Dox) for 48 h in combination with control or independent set of shRNA for G6PD (shG6PD-2). $NADP^+$/NADPH ratio (left) and protein abundance (right) were determined from breast cancer cells. In (**c, d**), data are means ± s.d. from n = 5 independent experiments. Statistical significances compared to control was denoted as *, $P < 0.05$; **, $P < 0.01$ by a two-tailed Student's t-test. Unprocessed original scans of blots are shown in Supplementary Fig. 11.

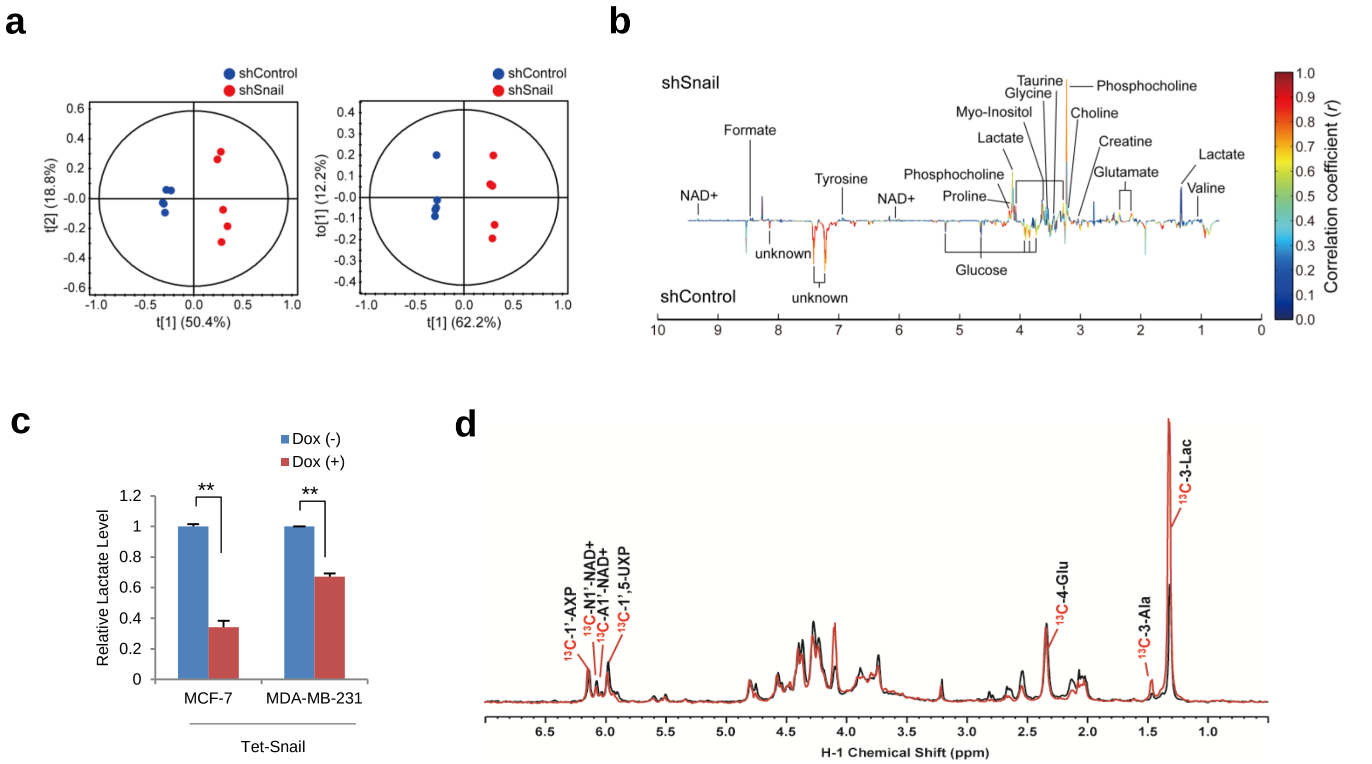

Supplementary Figure 3 EMT inducer Snail reprograms glucose metabolism. (**a**) Principle component analysis (PCA) score plot (left, R^2X = 0.692, Q^2 = 0.509) and orthogonal partial least-squares discriminant analysis (OPLS-DA) score plot (right, R^2X = 0.622, R^2Y = 998, Q^2 = 0.982) following knockdown of Snail (shSnail). (**b**) coefficient loading plot obtained by global profiling derived from ^{1}H NMR spectra of control (shControl) and Snail knockdown (shSnail). (**c**) The lactate level was measured in the indicated cells expressing doxycycline-inducible Snail. Data are means ± s.d. from n = 3 independent experiments. (**d**) A pair of representative 1D ^{1}H{^{13}C} HSQC NMR spectra show the changes in ^{13}C abundance which is represented by the intensity of ^{13}C-attached ^{1}H peaks of assigned metabolites in MDA-MB-231 cells expressing control shRNA (black) and Snail shRNA (red). Ala, alanine; Glu, glutamate; Lac, lactate; AXP, adenine nucleotides; UXP, uracil nucleotides. In (**a, c, d**), data are means ± s.d. from n = 5 independent experiments.

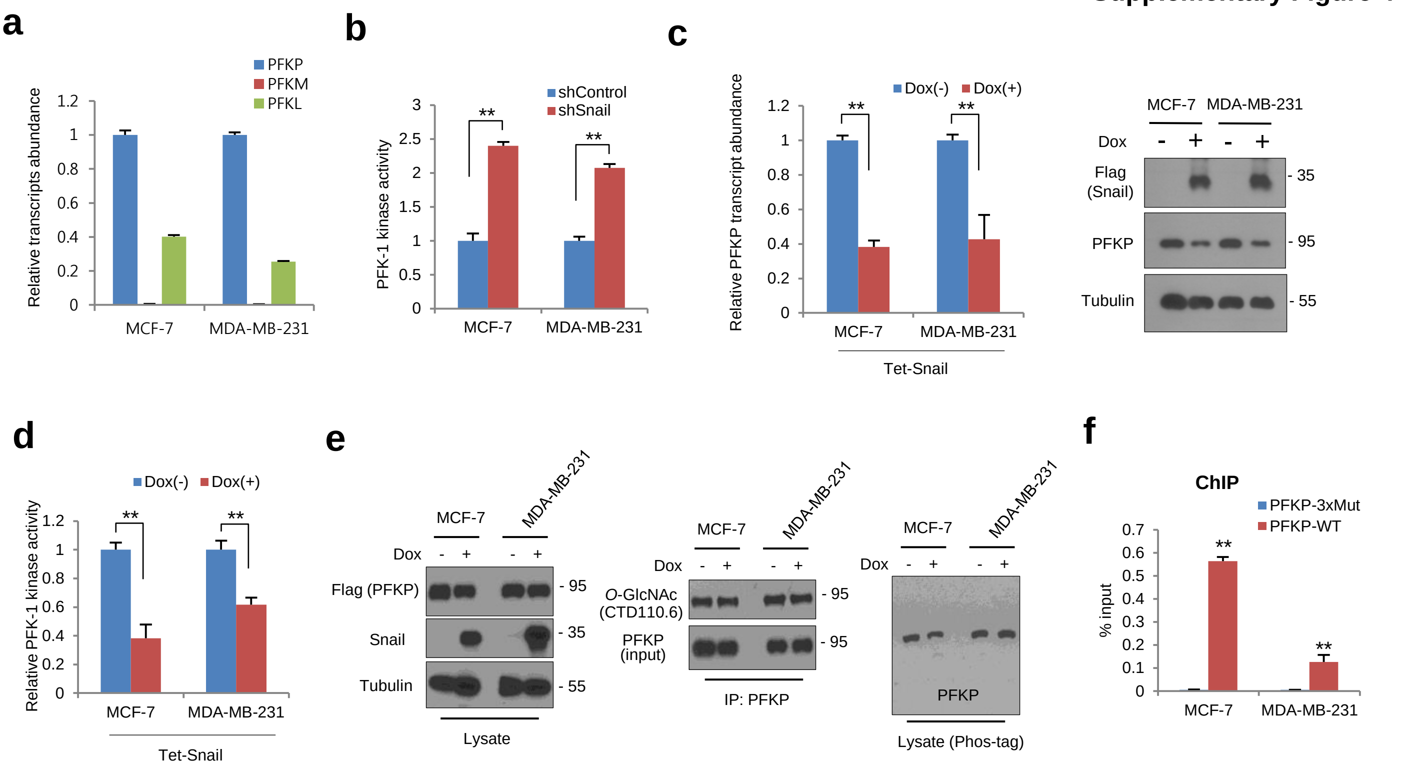

Supplementary Figure 4 Phosphofructokinase, platelet (PFKP) is a downstream target of Snail repressor in breast cancer cells. (**a**) Relative transcript abundance of PFK-1 isoforms was determined by quantitative PCR analysis in breast cancer cells. (**b**) PFK-1 kinase activity was determined following knock-down of Snail (shSnail) in breast cancer cells. (**c**) Relative transcript (left panel), protein (right panels) abundance and kinase activity (**d**) of PFKP following inducible expression of Snail in breast cancer cells. The cells expressing doxycycline-inducible Snail were treated with doxycycline (3 µg/ml) for 24 h, and the abundance of PFKP transcript and protein was measured by quantitative PCR (left panel) and immunoblot analysis (right panels). Representative blots are shown from at least two independent experiments. (**a-d**) Data are means ± s.d. from n = 3 independent experiments. (**e**) Posttranlational modification of PFKP according to Snail abundance. The PFKP was overexpressed without Snail (Dox-) or with Snail overexpression (Dox+), and the protein abundance of Snail and PFKP were determined from cell lystates (left panels). The lysate were subjected to immunoprecipitation with anti-PFKP antibody and the precipitate analysed by immunoblot with anti-O-GlcNAc and input PFKP (middle panels). Separately, the cell lysates were subjected to immunobloting for phosphorylation. Phos-tag denotes phosphorylation-tag gel used to resolve phosphorylated PFKP based on mobility shift. (**f**) Relative ChIP enrichment of wild type (wt) PFKP promoter compared to E-box mutant (3xMut). The cells were transfected with wt or 3xMut reporter vector, and then ChIP assays were performed with the Snail antibody. The precipitated samples were analyzed by quantitative PCR with specific primers for reporter vector; results are given as the percentage of input as means ± s.d. from 3 independent experiments. Unprocessed original scans of blots are shown in Supplementary Fig. 11.

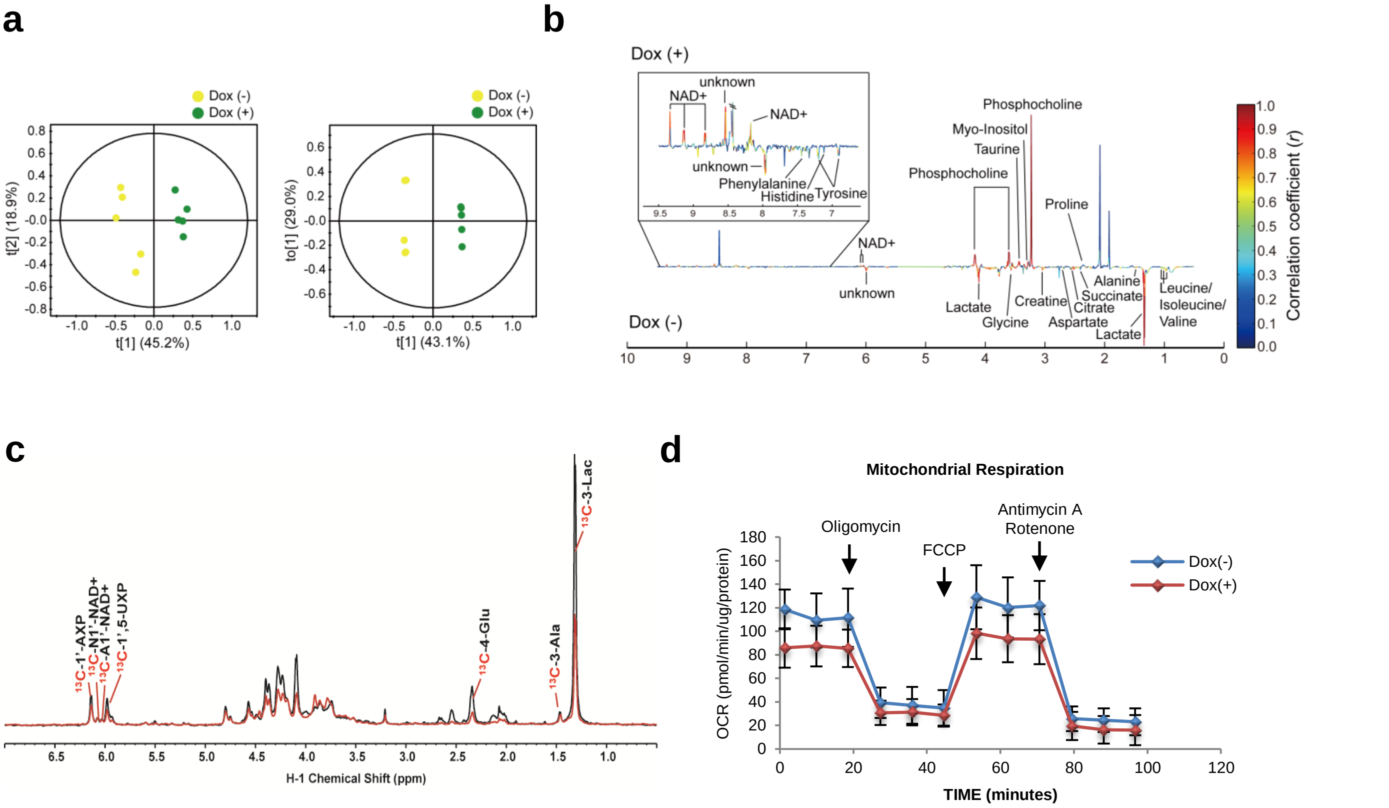

Supplementary Figure 5 PFKP is a gatekeeper of glucose flux into aerobic glycolysis. (**a**) Principle component analysis (PCA) score plot (left, R^2X = 0.641, Q^2 = 0.255) and orthogonal partial least-squares discriminant analysis (OPLS-DA) score plot (right, R^2X = 0.721, R^2Y = 1, Q^2 = 0.972) following shRNA-mediated PFKP knock-down (Dox+). **(b)** Coefficient loading plot obtained by global profiling derived from ^{1}H NMR spectra. (**c**) A pair of representative 1D ^{1}H{^{13}C} HSQC NMR spectra show the changes in ^{13}C abundance which is represented by the intensity of ^{13}C-attached ^{1}H peaks of assigned metabolites in MDA-MB-231 cells expressing control shRNA (black) and PFKP shRNA (red). Ala, alanine; Glu, glutamate; Lac, lactate; AXP, adenine nucleotides; UXP, uracil nucleotides. (**d**) Relative OCR normalized to protein abundance over time in control (Dox-, n = 5) and inducible knockdown of PFKP (Dox+, n = 5) cells.

Supplementary Figure 6

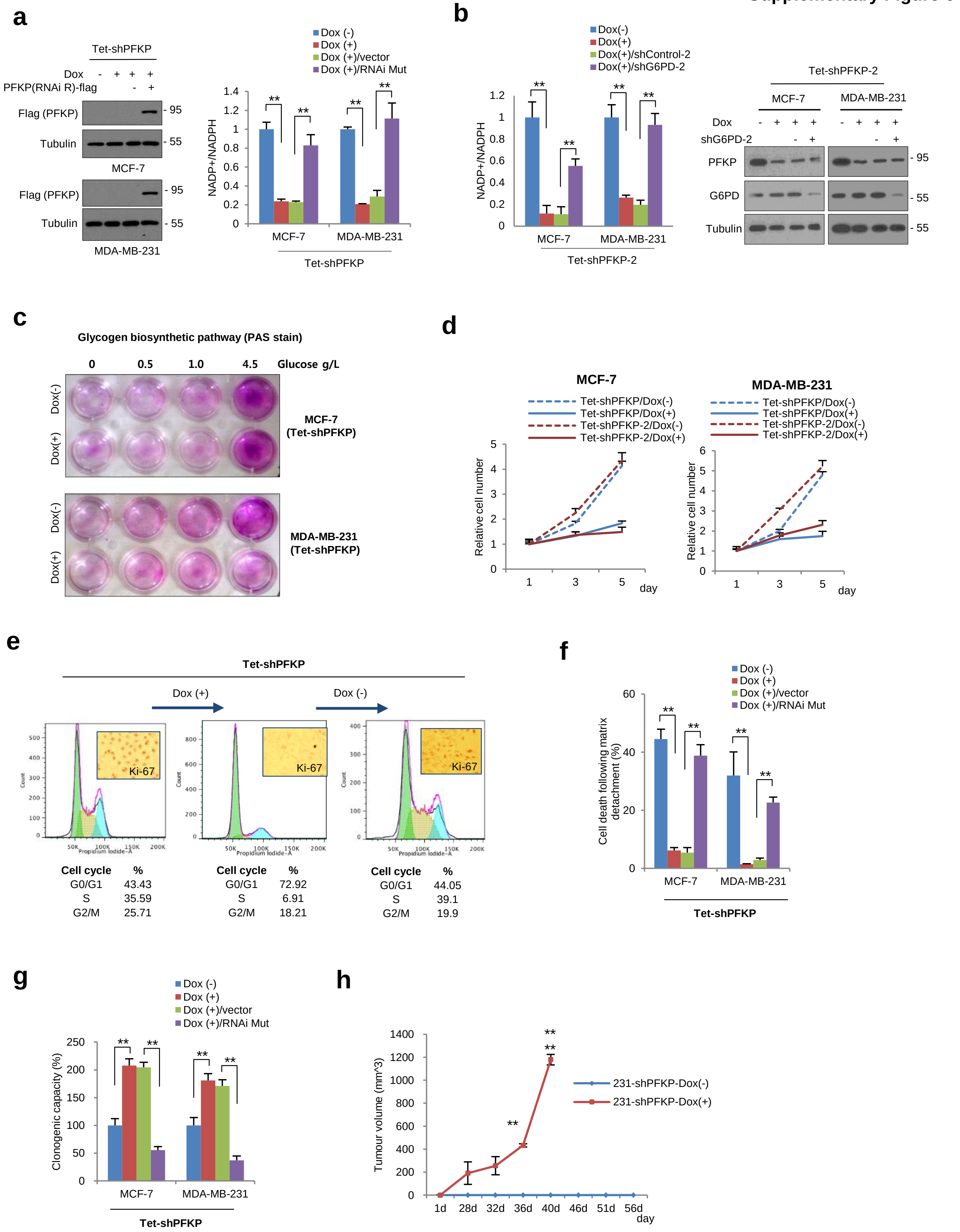

Supplementary Figure 6 PFKP function on glycogenesis, cell cycle and tumour initiation. (**a**) RNAi resistant PFKP rescued NADPH production by PFKP shRNA. Immunoblot analysis (left) of the breast cancer cells transfected with RNAi resistant expression vector as indicated following inducible knockdown of PFKP (Dox+). The transfection of shRNA resistant flag-tagged PFKP expression vector (RNAi-R) suppressed NADPH production (right) induced by shPFKP compared to vector control (-). (**b**) The $NADP^+$/NADPH ratio and immunoblot analysis (right) of indicated cells was measured following inducible knockdown of PFKP (Dox +) in combination with control shRNA or independent set of G6PD shRNA (shG6PD-2). NADPH data (**a**,**b**) are means ± s.d. from n = 3 independent experiments and two asterisks denote P < 0.01 by Student's *t*-test. (**c**) Loss of PFKP does not increase glycogen synthesis. The cells expressing doxycycline-inducible shPFKP were treated with negative control or doxycycline (shPFKP) and then cultured under various concentrations of glucose. The glycogen content was measured by Periodic acid-Schiff (PAS) stain. (**d**) Knock-down of PFKP induced growth arrest of breast cancer cells. Cell proliferation under normal culture condition was determined by MTT assay. Data are means ± s.d. from n = 5 independent experiments. (**e**) Flow cytometry analysis of the cell cycle in MDA-MB-231 cells expressing inducible PFKP shRNA. Doxycycline (3 μg/ml) was transiently treated for 48 h followed by refreshment of normal culture medium for 48 h. The cellular DNA contents were analyzed with flow cytometer after propidium iodide staining (0.05 mg/ml in PBS). Separately, the control (Dox -) and PFKP knockdown (Dox +) cells were immunostained with S phase marker Ki-67 (insets). (**f**) Cell death of breast cancer cells expressing inducible PFKP-shRNA (Dox +) and RNAi resistant PFKP expression vector after plating in detached (poly-HEMA-coated) plates. Cell death was measured by trypan blue exclusion. (**g**) Clonogenic assay of breast cancer cells expressing inducible PFKP-shRNA (Dox +) and RNAi resistant PFKP expression vector. Data (f & g) are means ± s.d. from n = 5 independent experiments and two asterisks denote P < 0.01 by Student's *t*-test. (**h**) Dynamic suppression of PFKP increased tumour initiating potential in vivo. Suboptimal number of tumour cells (1×10^5) was orthotopically injected into the mammary fat pads without extracellular matrix of either control (Dox-, n = 6) or transient knockdown of PFKP (Dox+, n = 6). Recipient mice received either Dox (red) or vehicle (blue) intraperitoneally during initial period of tumour inoculation as described in Fig. 5f. Tumour initiation and growth was measured twice a week; results are shown as means and s.e.m. Statistical significance was determined by Mann-Whitney test. Two asterists denote P < 0.01. Unprocessed original scans of blots are shown in Supplementary Fig. 11.

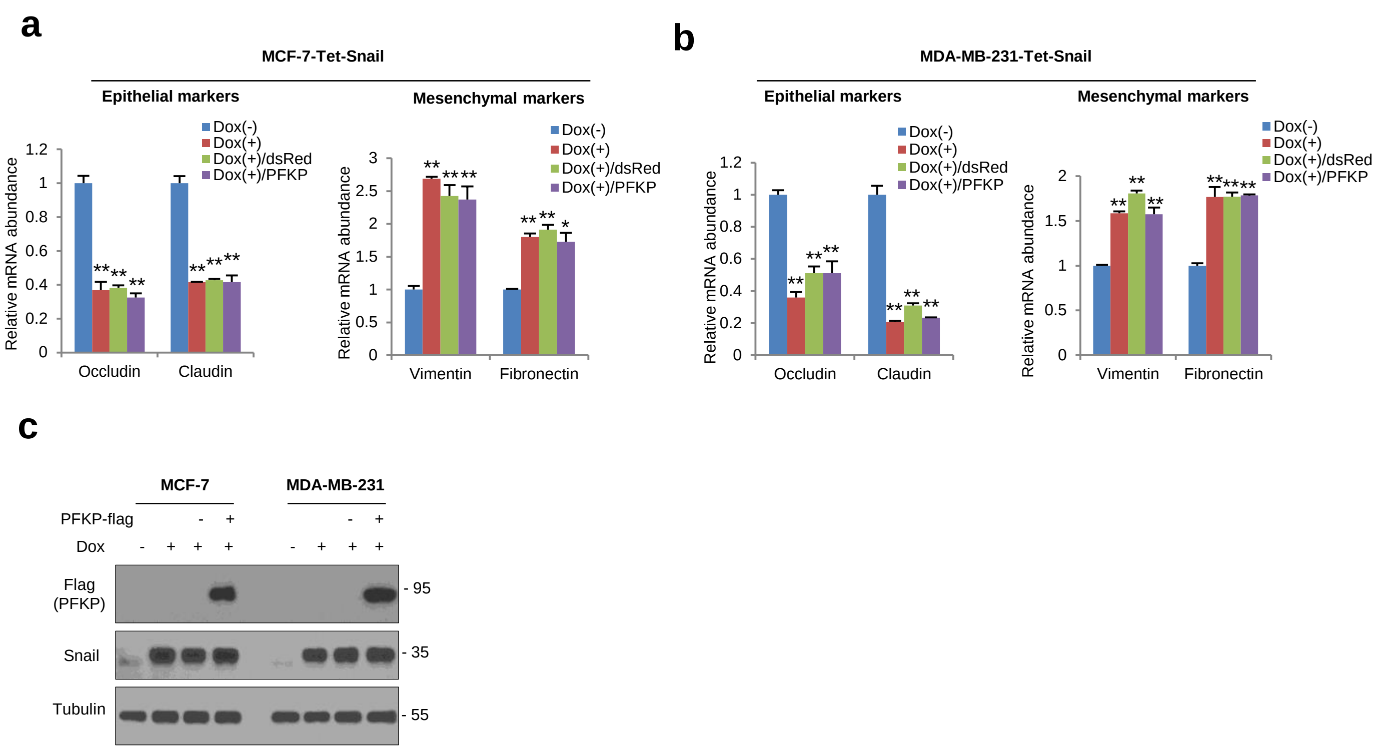

Supplementary Figure 7 PFKP function on phenotypic conversion of EMT. (**a, b**) The relative mRNA transcript abundance of EMT responsive genes in MCF-7 (**a**) and MDA-MB-231 cells (**b**). Note that EMT induction by overexpression of Snail was not rescued by PFKP. Data are means ± s.d. from n = 3 independent experiments. (**c**) Immunoblot analysis for overexpressed PFKP and Snail in breast cancer cells for the above qPCR analysis. Images are representative of two independent experiments. Two asterists denote $P < 0.01$ by Student's *t*-test. Experiments **a**, **b** are representative and were replicated from at least two independent experiments. Unprocessed original scans of blots are shown in Supplementary Fig. 11.

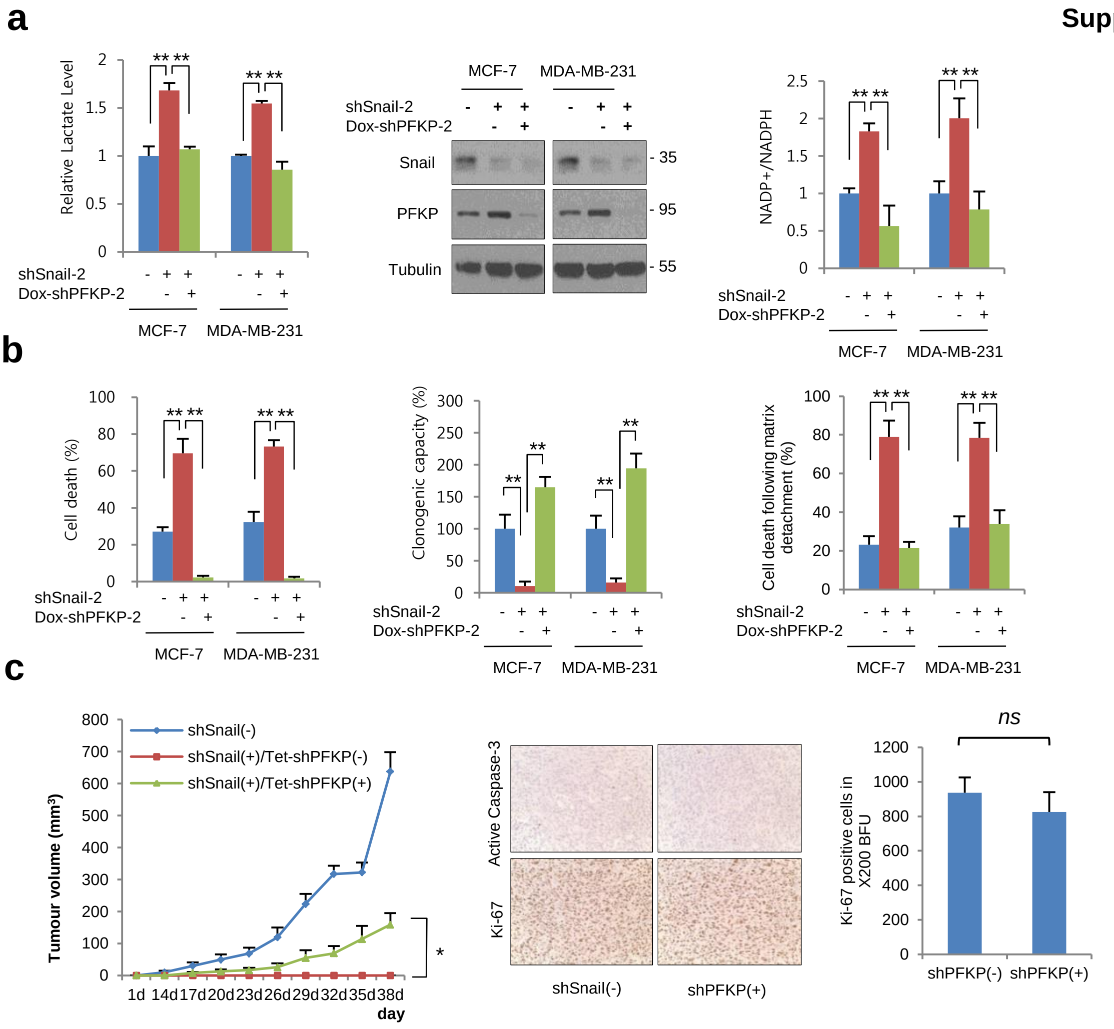

Supplementary Figure 8 Suppression of PFKP rescued glucose reprogramming and cancer cell survival induced by loss of Snail. (**a**) Independent set of shRNA rescue experiment in Fig. 6. Inducible knockdown of PFKP (shPFKP-2) rescued lactate production (left), immunoblot (upper middle), NADPH production (upper right). Data are means ± s.d. from n = 3 independent experiments. (**b**) Inducible knockdown of PFKP (shPFKP-2) rescued cell death under glucose starvation (left) and clonogenic capacity of the starved cells (middle) and cell death after matrix detachment (right) induced by Snail knockdown (shSnail-2). Data are means ± s.d. from n = 5 independent experiments. Two asterisks denote P < 0.01 by Student's *t*-test. Experiments (**b**) are representative and were replicated from at least two independent experiments. (**c**) MDA-MB-231 cells (1 x 10^6, n = 4) were orthotopically injected into the mammary fat pads without extracellular matrix of either (1) control [shSnail(-)], or (2) knockdown of Snail without transient knockdown of PFKP [shSnail(+)/Tet-PFKP(-)], or (3) knockdown of Snail with transient knockdown of PFKP [shSnail(+)/Tet-PFKP(+)]. Transient knockdown with inducible PFKP shRNA was performed as described in Fig. 5f. Tumour initiation and growth was measured twice a week; results are shown as means and s.e.m. (left). Two asterists denote P < 0.01 by Mann-Whitney test. Representative images of immunohistochemical staining (middle) of active caspase-3 (cell death) and Ki-67 (proliferation) from tumour tissue of xenograft mice with control (group 1, shSnail-) or with knockdown of PFKP (group 3, shPFKP-). There is no statistical difference of proliferation index by Student's *t*-test (right). Unprocessed original scans of blots are shown in Supplementary Fig. 11.

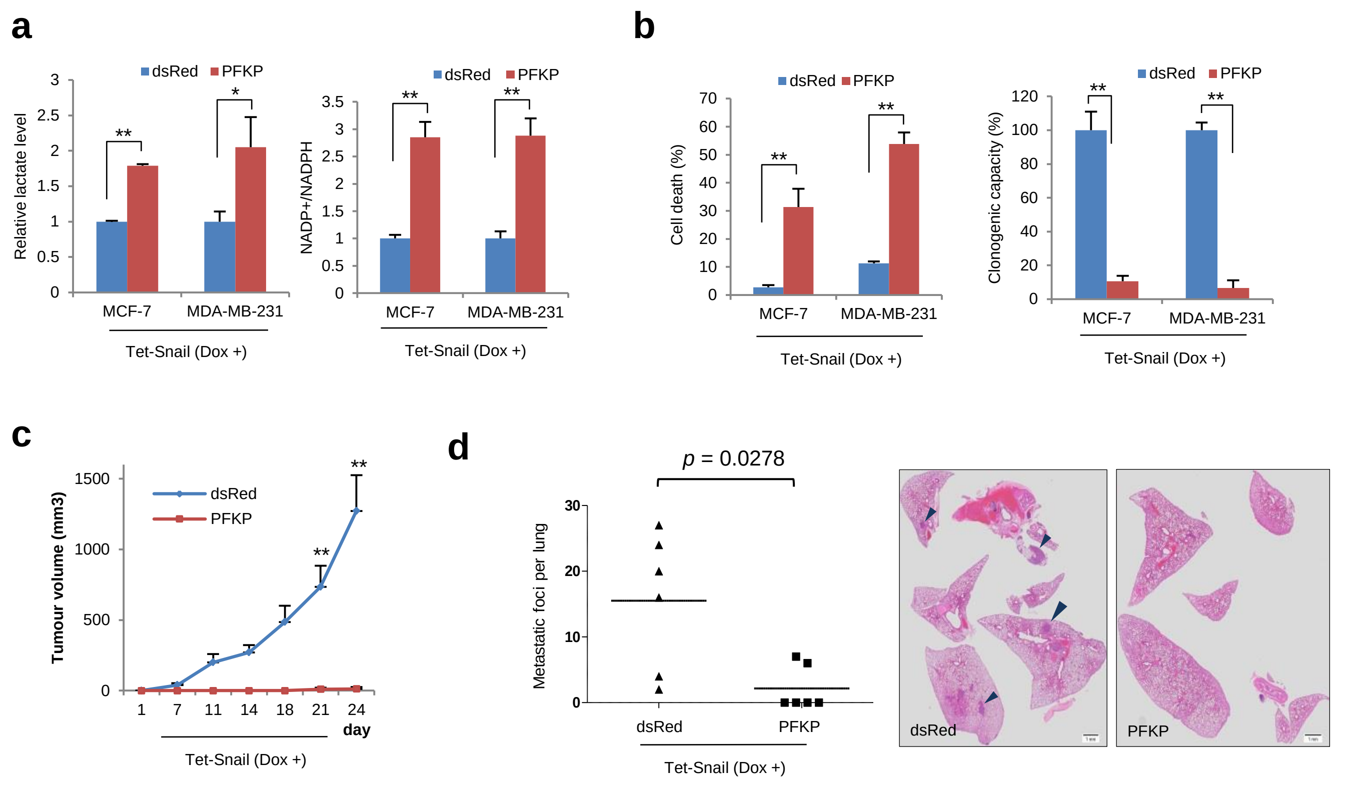

Supplementary Figure 9 Increased glycolytic activity by overexpression of PFKP relieved Snail-mediated metabolic reprogramming in vitro and in vivo. (**a**) Lactate production (left) and NADPH production (right) by overexpression of PFKP under induction of Snail in breast cancer cells. Data are means ± s.d. from n = 3 independent experiments of biological replicates. (**b**) Cell death under glucose starvation (left) and clonogenic capacity of the starved cells (right). Data are means ± s.d. from n = 5 independent experiments of biological replicates. Two asterisks denote P < 0.01 by Student's *t*-test. (**c**) MDA-MB-231 cells (5 x 10^5, n = 6) were orthotopically injected into the mammary fat pads without extracellular matrix of either control (dsRed) or overexpression of PFKP (PFKP). Snail was transiently overexpressed as described in Fig. 5f. Tumour initiation and growth was measured twice a week; results are shown as means and s.e.m. Two asterisks denote P < 0.01 by Mann-Whitney test. (**d**) Lung metastasis by tail vein xenograft of Snail-expressing MDA-MB-231-D3H2LN cells. 1 x 10^5 cells either of control (dsRed, n = 6) or of overexpression of PFKP (PFKP, n = 6) were injected intravenously into immunodeficient mice. The number of lung metastatic nodules at day 28 was counted under microscopic examination (left). Statistical significance was determined by Mann-Whitney test. Whole-field images of representative lungs that showed median metastatic value for each group (right). Arrows indicate metastatic tumour foci in mouse lung and scale bars are 1 mm. In vivo experiments (**c**, **d**) are representative and were replicated from at least two independent experiments. Unprocessed original scans of blots are shown in Supplementary Fig. 11.

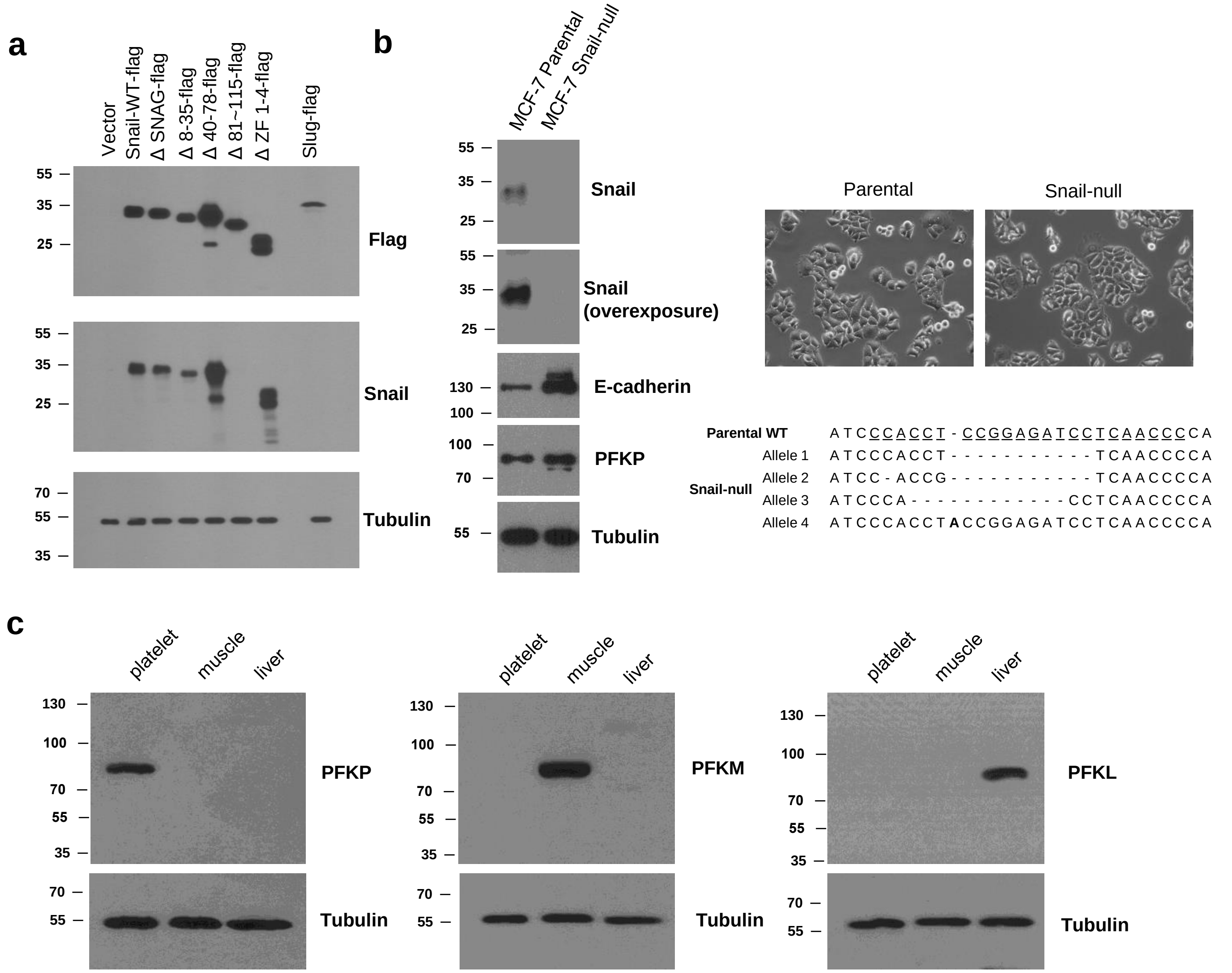

Supplementary Figure 10 Validation of antibodies used in this study. (**a**) Mapping of anti-Snail antibody recognition motif with serially deleted Snail and Slug expression vectors. The flag-tagged expression vectors were transfected into 293 cells, and antibody recognition motif was examined with anti-Flag and anti-Snail antibody (left panels). (**b**) Snail-knockout MCF-7 cells were generated by CRISPR-Cas9 technology (Snail-null), and 50 µg of whole cell lysates from parental and isogenic Snail-null MCF-7 were subjected to Snail immunoblot analysis and luminescence was overexposed overnight. Phase contrast pictures of parental and isogenic Snail-null MCF-7 cell (right upper), and genomic sequences Snail exon 1 of Snail-null MCF-7 cells (right lower). The target genomic sequences of single guide RNA were underlined, and copy number variation of Snail was examined from Cell Line Synopsis (https://cansar.icr.ac.uk/cansar/cell-lines/). (**c**) Mouse platelet, muscle and liver tissue were used for specific antibodies against PFK-1 isoforms. Thirty µg of each mouse tissue lysate were subjected to immunoblot analysis with each antibody. The loading control with tubulin antibody was performed with the same membrane following de-blotting of PFK-1 antibodies.

STR Profile Results

Sample	D8S1179	D21S11	D7S820	CSF1PO	D3S1358	TH01	D13S317	D16S539
**MCF-7 (control)**	**10,14**	**30**	**8,9**	**10**	**16**	**6**	**11**	**11,12**
MCF-7	10,14	30	8,9	10	16	6	11,12	11,12
**MDA-MB-231 (control)**	**13**	**30,33.2**	**8,9**	**12,13**	**16**	**7,9.3**	**13**	**12**
MDA-MB-231	13	30,33.2	8	13	16	7,9.3	13	12
**HEK293T (control)**	**12,14**	**28,30.2**	**11,12**	**11,12**	**15,17**	**7,9.3**	**12,14**	**9,13**
HEK293T	12,15	27,18,30.2	10,11	11,12	15,17	7,9.3	11,12,13,14	9,12
**HCT116 (control)**	**12,14**	**29,30**	**11,12**	**7,10**	**12,19**	**8,9**	**10,12**	**11,13**
HCT116	13,14	29,30	11,12	7,10	12,19	8,9	10,12	11,13
**SW480 (control)**	**13**	**30,30.2**	**8**	**13,14**	**15**	**8**	**12**	**13**
SW480	13	30,30.2	8	13,14	15	8	12	13
**DLD-1 (control)**	**15**	**29,32.2**	**10,12**	**11,12**	**17**	**7,9.3**	**8,11**	**12,13**
DLD-1	15	29,32.2	10,12	12	17	7,9.3	8,11	12,13

Sample	D2S1338	D19S433	Vwa	TPOX	D18S51	Amelogenin	D5S818	FGA
**MCF-7 (control)**	**21,23**	**13**	**14,15**	**9,12**	**14**	**X**	**11,12**	**23,24,25**
MCF-7	21,23	13,14	14,15	9,11,12	14	X	12	23,25
**MDA-MB-231 (control)**	**20,21**	**11,14**	**15,18**	**8,9**	**11,16**	**X**	**12**	**22,23**
MDA-MB-231	20,21	11	15	8,9	11,16	X	12	22,23
**HEK293T (control)**	**19**	**15,18**	**16,19**	**11**	**17,18**	**X**	**8,9**	**23**
HEK293T	19	16,17	16,19,20	11	17,18	X	8,10	22,23
**HCT116 (control)**	**16**	**12,13**	**17,22**	**8**	**17**	**X,Y**	**10,11**	**18,23**
HCT116	16	12,13	17,22	8	17	X	10,11	18,23
**SW480 (control)**	**17,24**	**13**	**16**	**11**	**12,13**	**X**	**13**	**24**
SW480	17,24	13	16	11	13	X	13	24
**DLD-1 (control)**	**17,25**	**14,16**	**18,19**	**8,11**	**11,17**	**X,Y**	**13**	**22**
DLD-1	17,25	14,16	18,19	8,11	11,17	X,Y	13	22

Supplementary Figure 11 Short tandem repeat (STR) profiling of cell lines used in this study. DNA fingerprinting of cell line was performed by AmplFLSTR PCR amplification kit (Applied Biosystem) and analyzed by GeneMapper ver 5.0 (Korean Cell Line Bank). Following the guideline of the American Tissue Culture Collection Standards Development Organization Workgroup, the test included at least 8 STR loci (TH01, TPOX, Vwa, CSF1PO, D16S539, D13S317, D5S818 and Amelogenin). Upper (bold) and lower lanes represent ATCC standard and results of cell lines fingerprinting used in this study.

Supplementary Figure 12 Uncropped images of all the western blot data

Fig. 1a

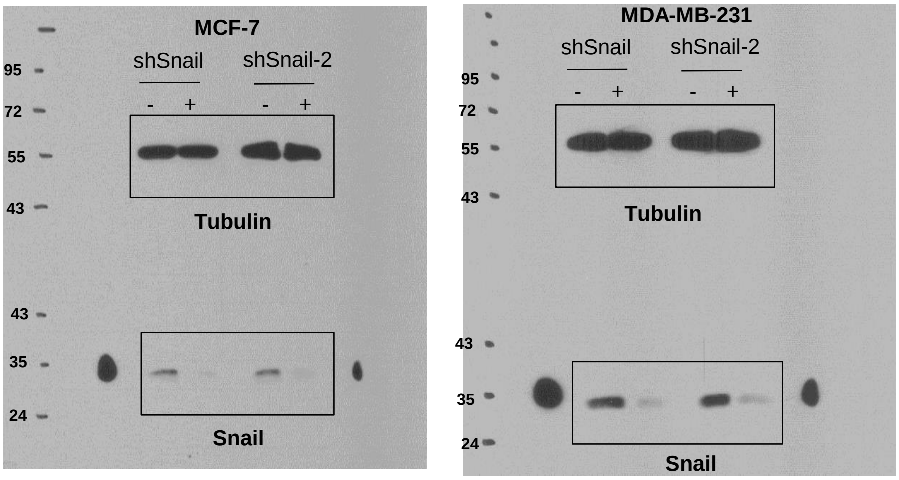

Fig. 1h

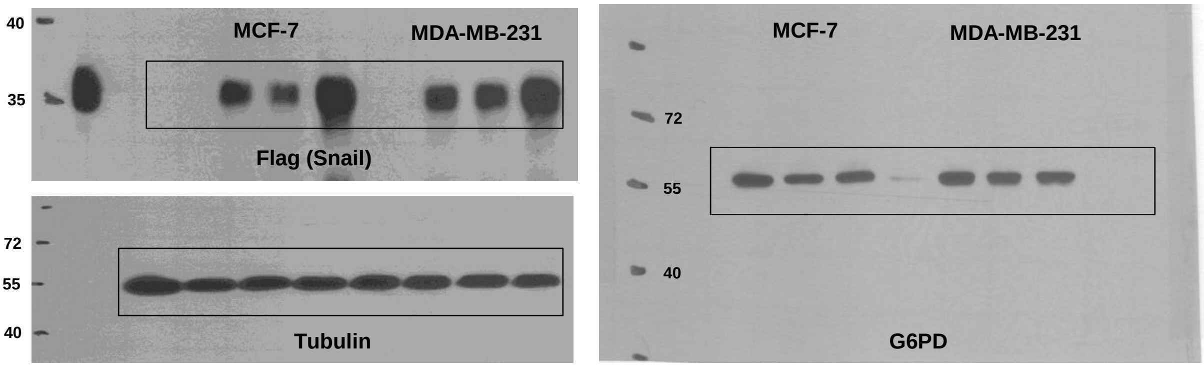

Fig. 3a

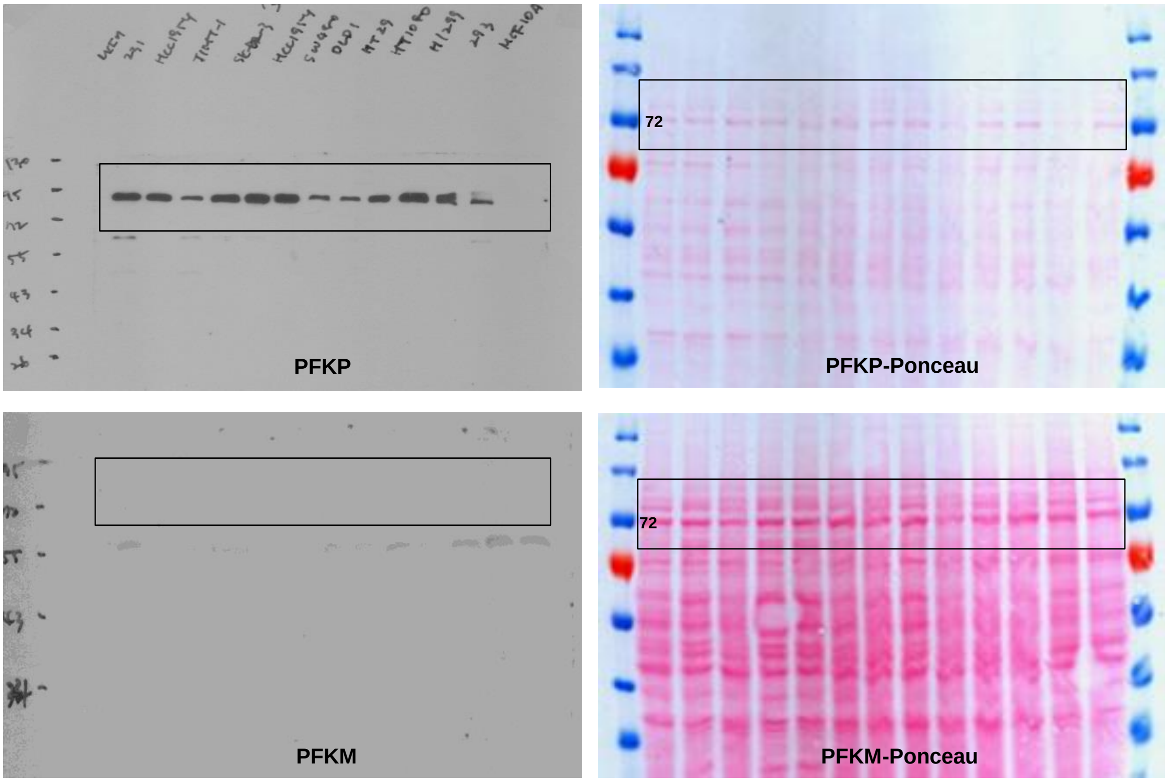

Supplementary Figure 12 Continued

Fig. 3a (continued)

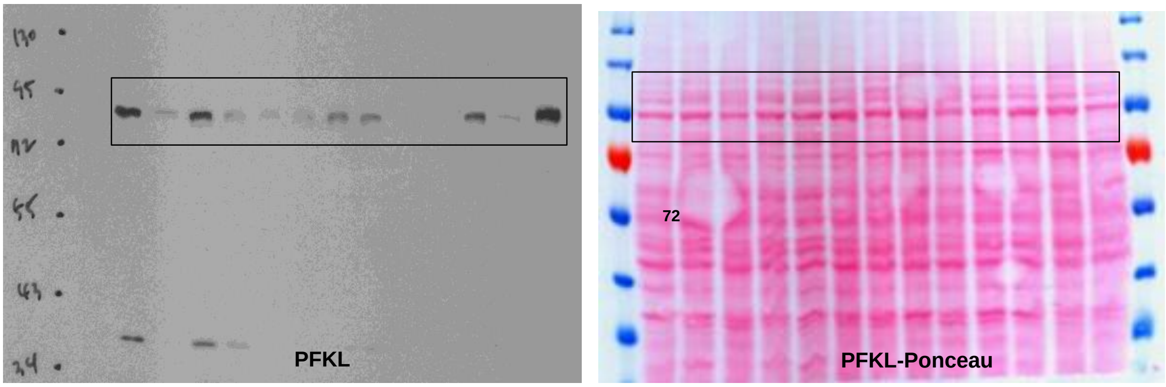

Fig. 3b

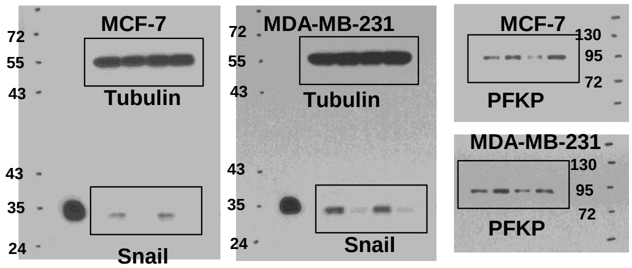

Fig. 3e

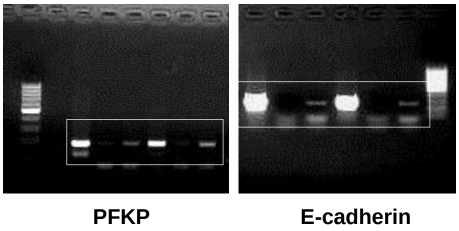

Figure 4d

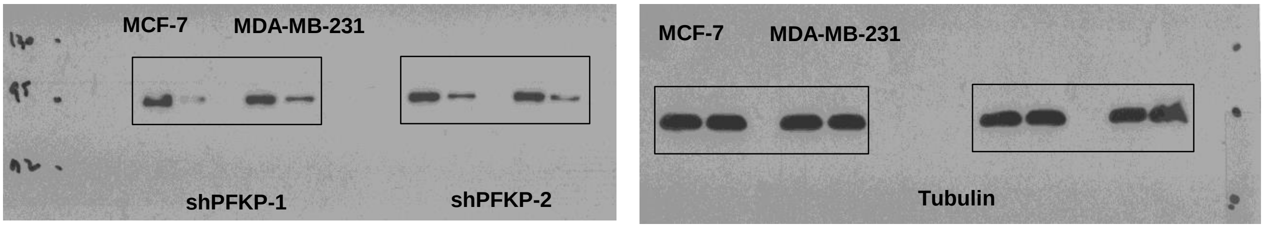

Fig. 5a

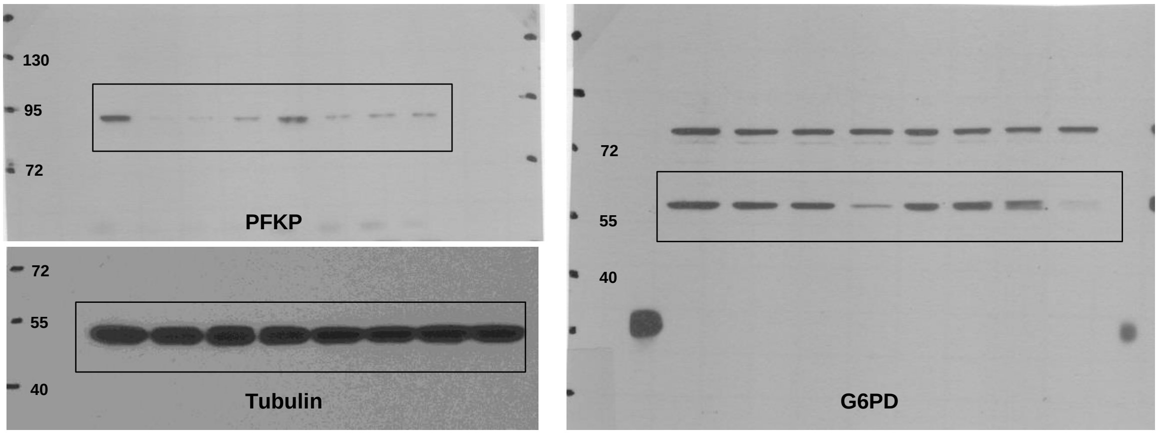

Supplementary Figure 12 Continued

Fig. 6a

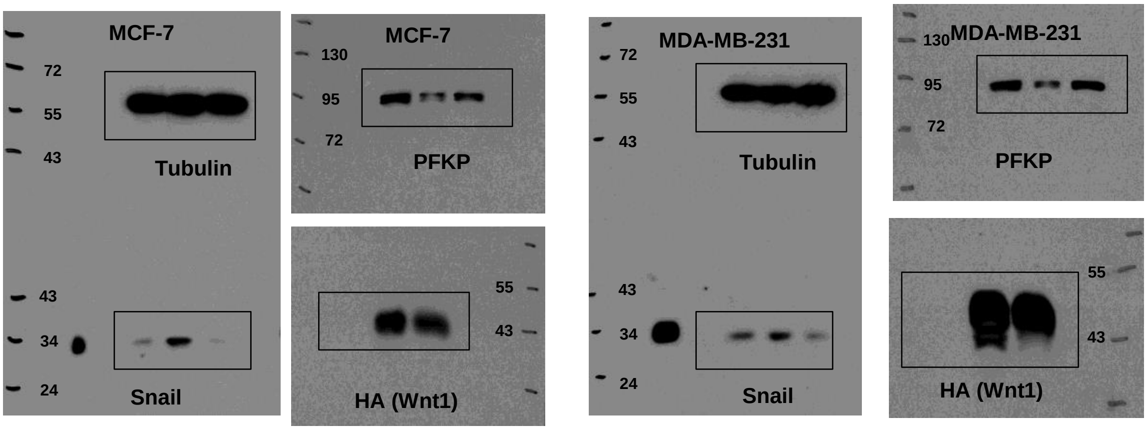

Fig. 7a

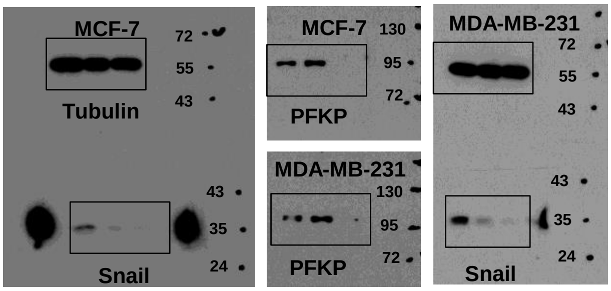

Figure S1b

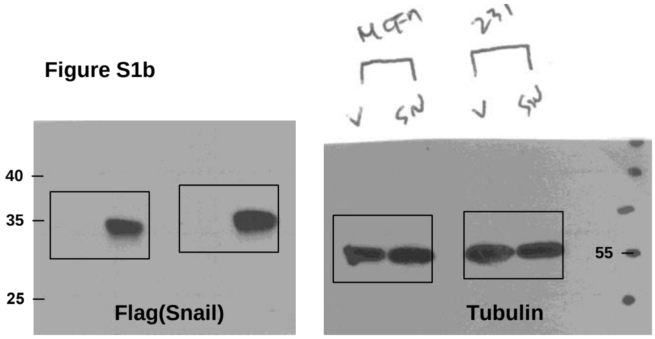

Figure S1e

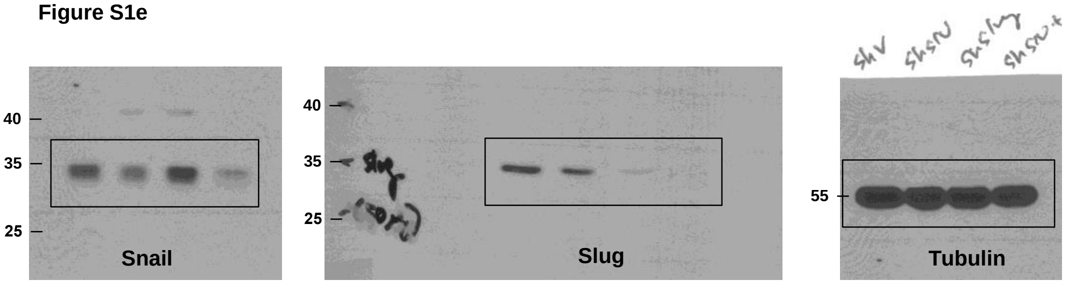

Supplementary Figure 12 Continued

Supplementary Fig. 2d

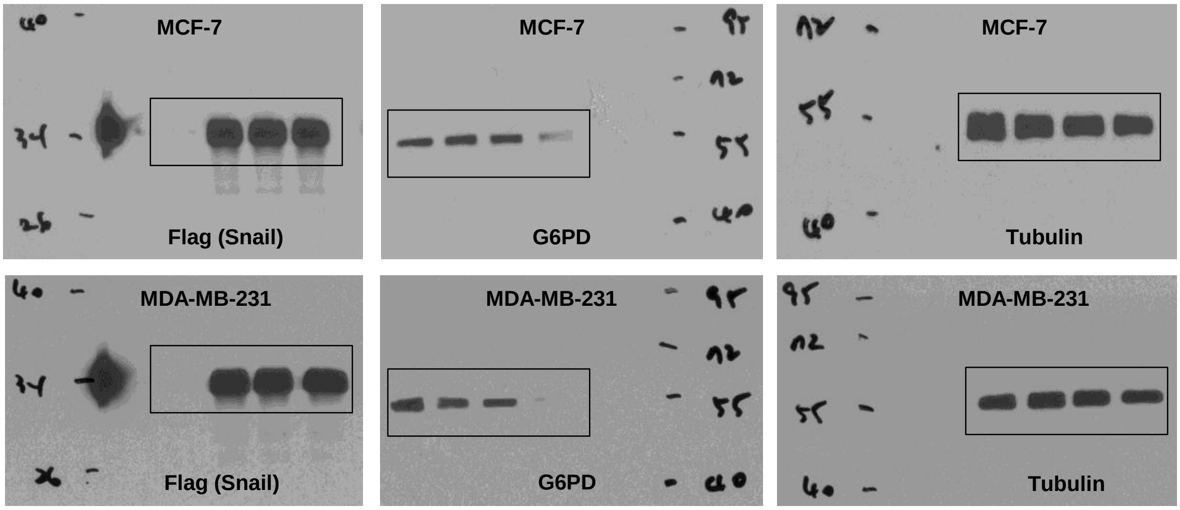

Supplementary Fig. 4c

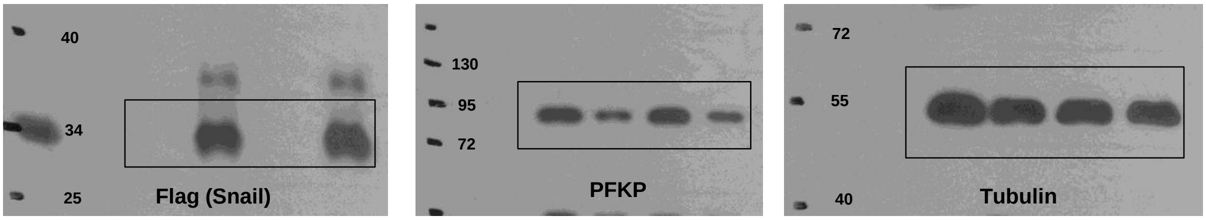

Supplementary Fig. 4e

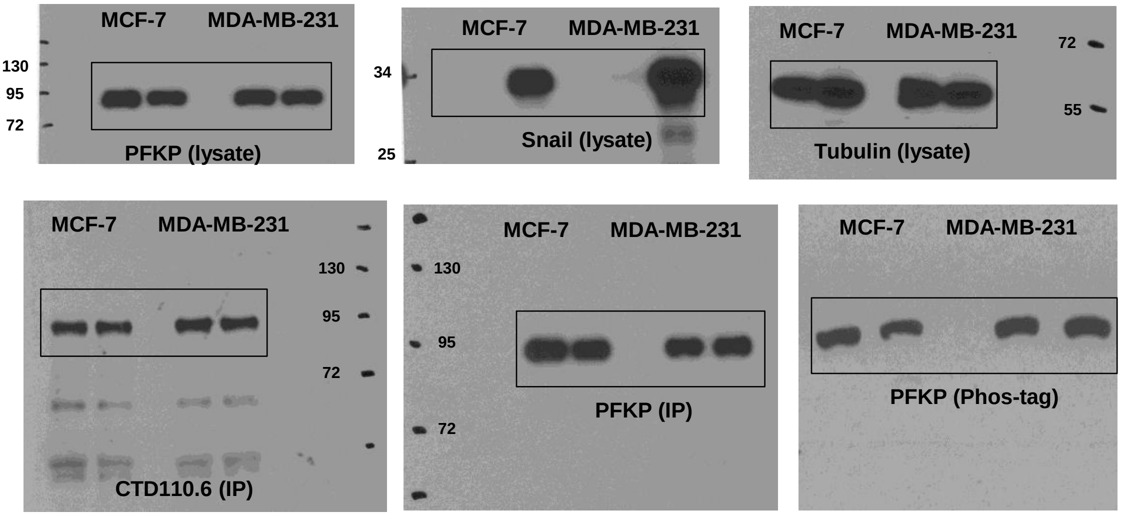

Supplementary Figure 12 Continued

Supplementary Fig. 6a

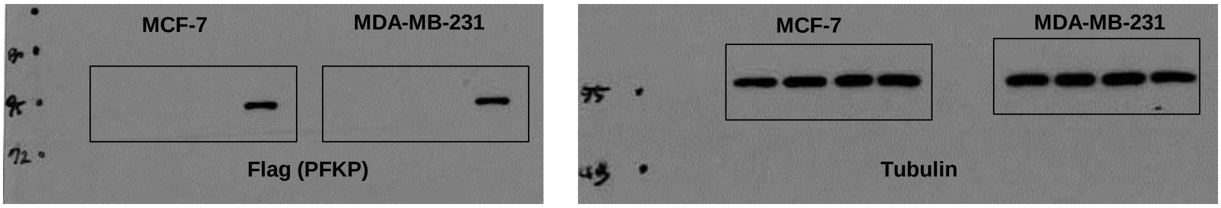

Supplementary Fig. 6b

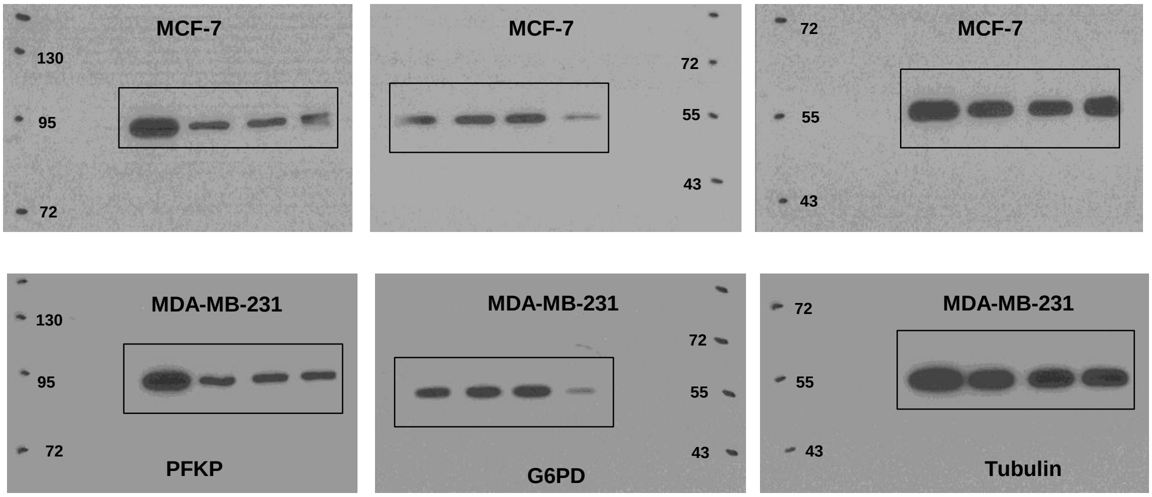

Supplementary Fig. 7c

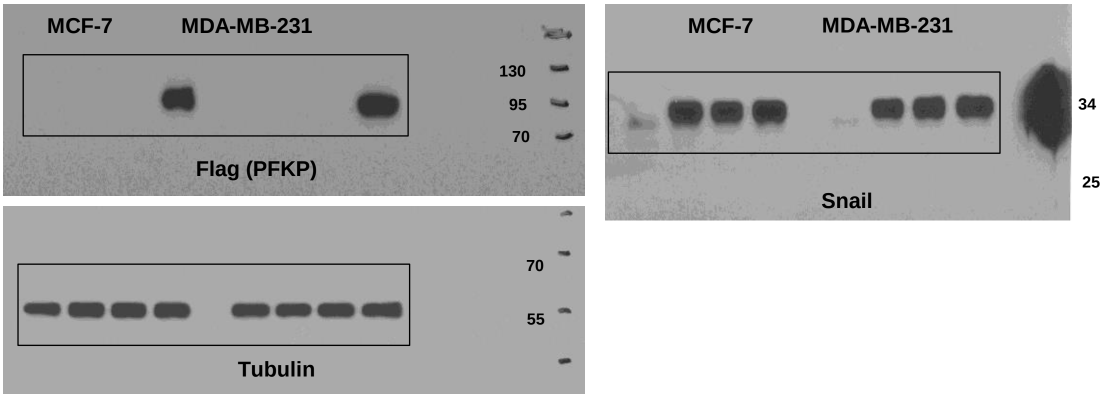

Supplementary Fig. 8a

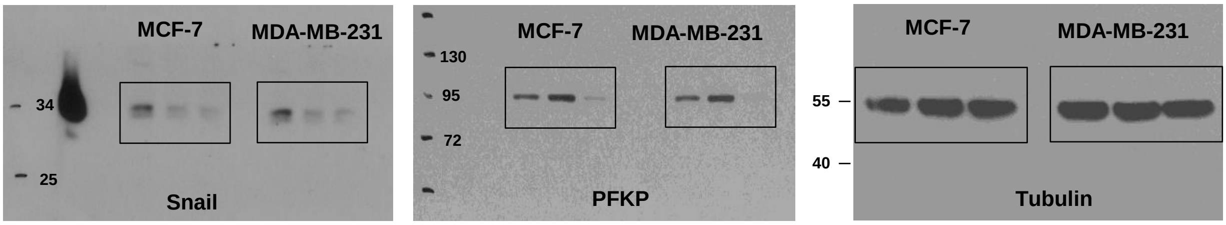

Supplementary Information

Primer sequences for qPCR analysis

Gene	Forward	Reverse
PFKP	5’- cggagttcctggagcacctctc	5’-aagtacaccttggcccccacgta
PFKM	5’-gagtgacttgttgagtgacctccagaaa	5’-cacaatgttcaggtagctggacttcg
PFKL	5’-ggcatttatgtgggtgccaaagtc	5’-cagttggcctgcttgatgttctca
Occludin	5’-cggtctaggacgcagcagat	5’-aagaggcctggatgacatgg
Claudin	5’-ggctgctttgctgcaactgtc	5’-gagccgtggcaccttacacg
Vimentin	5’-tttttccagcaagtatccaacc	5’-gagttttccaaagatttattgaa
Fibronectin	5’-caggatcacttacggagaaacag	5’-gccagtgacagcatacacagtg
GAPDH	5’-atgggtgtgaaccatgagaag	5’-agttgtcatggatgaccttgg
